# 

**Published:** 1898-09

**Authors:** 


### Southern Medical Record

A Monthly Journal of Medicine and Surgery.

VOL. XXVIII. ATLANTA, GA., SEPTEMBER, 1898. No. 9

LIBRARY SURGEON GENERAL'S OFFICE SEP.-23-1898

BERNARD WOLFF, M. D., EDITOR.

ASSOCIATE EDITORS:

A. W. GRIGGS, M. D. J. McFADDEN GASTON, M. D.

J. McFADDEN GASTON, Jr., M. D. WILLIS F. WESTMORELAND, M. D.

B. M. ZETTLER, BUSINESS MANAGER.

Entered at the Post-office at Atlanta, Ga., as Second-class Matter.

